# 4-Fluoro­anilinium tetra­chloridoferrate(III) 18-crown-6 clathrate

**DOI:** 10.1107/S160053681002009X

**Published:** 2010-06-05

**Authors:** Jia-Zhen Ge, Min-Min Zhao

**Affiliations:** a Ordered Matter Science Research Center, College of Chemistry and Chemical Engineering, Southeast University, Nanjing 211189, People’s Republic of China

## Abstract

The reaction of 4-fluoro­aniline hydro­chloride, 18-crown-6 and ferric chloride in methano­lic solution yields the title compound, (C_6_H_7_FN)[FeCl_4_]·C_12_H_24_O_6_, which has an unusual supramolecular structure. N—H⋯O hydrogen-bonding inter­actions between the NH_3_^+^ substituents of the 4-fluoro­anilinium cations and the O atoms of the crown ether mol­ecules result in a rotator–stator-like structure.

## Related literature

For a related 18-crown-6 clathrate, see: Fender *et al.* (2002 ▶). For the ferroelectric properties of selected transition metal complexes, see: Fu *et al.* (2007 ▶); Ye *et al.* (2009 ▶); Zhang *et al.* (2009 ▶).
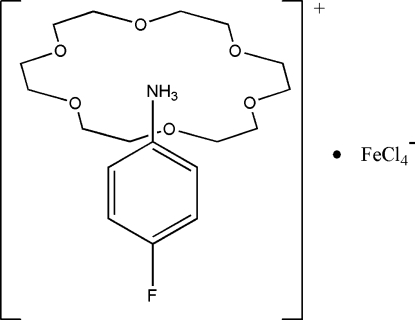


## Experimental

### 

#### Crystal data

(C_6_H_7_FN)[FeCl_4_]·C_12_H_24_O_6_
*M* *_r_* = 574.09
Monoclinic, 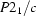

*a* = 11.45 (1) Å
*b* = 24.14 (2) Å
*c* = 9.719 (9) Å
β = 96.82 (2)°
*V* = 2667 (4) Å^3^
*Z* = 4
Mo *K*α radiation
μ = 1.00 mm^−1^
*T* = 293 K
0.20 × 0.20 × 0.20 mm

#### Data collection

Rigaku SCXmini diffractometer
Absorption correction: multi-scan (*CrystalClear*; Rigaku, 2005 ▶) *T*_min_ = 0.818, *T*_max_ = 0.818
26978 measured reflections
6039 independent reflections
3173 reflections with *I* > 2σ(*I*)
*R*_int_ = 0.068

#### Refinement

*R*[*F* ^2^ > 2σ(*F* ^2^)] = 0.078
*wR*(*F* ^2^) = 0.271
*S* = 1.07
6039 reflections
281 parameters
H-atom parameters constrained
Δρ_max_ = 0.49 e Å^−3^
Δρ_min_ = −0.35 e Å^−3^

### 

Data collection: *CrystalClear* (Rigaku, 2005 ▶); cell refinement: *CrystalClear*; data reduction: *CrystalClear*; program(s) used to solve structure: *SHELXS97* (Sheldrick, 2008 ▶); program(s) used to refine structure: *SHELXL97* (Sheldrick, 2008 ▶); molecular graphics: *SHELXTL* (Sheldrick, 2008 ▶); software used to prepare material for publication: *PRPKAPPA* (Ferguson, 1999 ▶).

## Supplementary Material

Crystal structure: contains datablocks I, global. DOI: 10.1107/S160053681002009X/im2203sup1.cif

Structure factors: contains datablocks I. DOI: 10.1107/S160053681002009X/im2203Isup2.hkl

Additional supplementary materials:  crystallographic information; 3D view; checkCIF report

## Figures and Tables

**Table 1:** Hydrogen-bond geometry (Å, °)

*D*—H⋯*A*	*D*—H	H⋯*A*	*D*⋯*A*	*D*—H⋯*A*
N1—H1*C*⋯O4^i^	0.89	1.98	2.868 (6)	176
N1—H1*D*⋯O6^i^	0.89	2.04	2.924 (6)	173
N1—H1*E*⋯O2^i^	0.89	1.98	2.840 (6)	162

Symmetry code: (i) 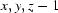.

**Fig. 1.**
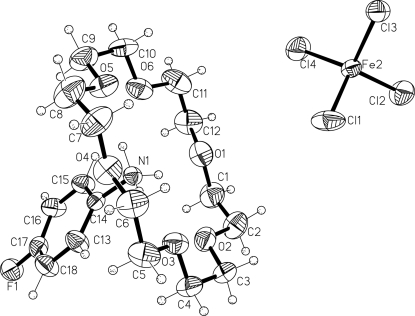
The molecular structure of the title compound, with the atomic numbering scheme. Displacement ellipsoids are drawn at the 30% probability level.

**Fig. 2.**
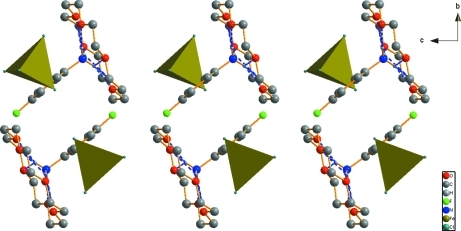
A view of the packing of the title compound, stacking along the a axis. Dashed lines indicate hydrogen bonds.

## supplementary crystallographic information

### Comment

Crown ethers have attracted much attention because of their ability to form non-covalent, H-bonding complexes with ammonium cations both in solid and in solution (Fender *et al.* 2002). Both the size of the crown ether and the nature of the ammonium cation (-NH_4_^+^, RNH_3_^+^, *etc*) can influence the stoichiometry and stability of these host-guest complexes. The host molecules combine with the guest species by intermolecular interactions, and if the host molecule possess some specific sites (by chelate effect), it is easy to realise high selectivity in ion or molecular recognitions.18-crown-6 have the highest affinity for ammonium cation RNH_3_^+ ^and most studies of 18-crown-6 and its derivatives invariably showed a 1:1 stoichiometry with RNH_3_^+^ cations.

In continuation of our investigations on ferroelectric phase transitions materials the dielectric permittivity of the title compound was tested (Fu *et al.* 2007; Ye *et al.* 2009; Zhang *et al.* 2009). The title compound shows no dielectric anomalies with values of 6-8 and 7-10 in the temperature ranges from 80 to 300 K and 300 K to 400 K (below the compound melting point 433 K), respectively. These findings suggest that the compound should exhibit no distinct phase transition within the measured temperature range.

The title compound crystallizes in the *P*2_1_/c space group. The asymmetric unit of the title compound is composed of a cationic [(C_6_H_4_FN_3_) (18-Crown-6)]^+^ moiety and one isolated anionic [FeCl_4_]^-^ (Fig 1). The protonated *p*-fluoroanilinium [C_6_H_4_FNH_3_]^+^ and 18-crown-6 form a superamolecular rotator-stator-like structure by forming N—H···O hydrogen bonds between the -NH_3_^+^ substitutents of the cations and oxygen atoms of crown ethers. Intramolecular N—H···O hydrogen distances within the usual range: 2.950 (6) and 2.840 (6) Å. The crown ring is slight distorted. The six oxygen atoms of the crown ether lie approximately in a plane. The C—N bonds of [C_6_H_4_FNH_3_]^+^ are almost perpendicular to the mean oxygen plane.

The typical Fe—Cl bond lengths in the tetrahedral coordinate anion [FeCl_4_]^-^ are within 2.170 (3)-2.184 (2) Å. The Cl—Fe—Cl bond angles indicate little distortion from a regular tetrahedron [spread of values 108.3 (1)-110.7 (1)°].

Fig. 2 shows a view down the *a* axis. An alternate arrangement of cation and anion layers is observed along the *c* axis, a couple of head-to-head rotator-stator cations and an anion [FeCl_4_]^-^ along the *b* axis. No significantly short intermolecular hydrogen bond was observed.

### Experimental

*p*-F-C_6_H_4_-NH_2 _× HCl (2 mmol, 0.295 g) and 18-crown-6 (2 mmol, 0.528 g) were dissolved in methanol. After addition of ferric chloride (2 mmol, 0.54 g) in concentrated hydrochloric acid, a precipitate (yield is about 95%) was formed, filtered and washed with a small amount of methanol. Single crystals suitable for X-ray diffraction analysis were obtained from slow evaporation of methanol and DMF (*v/v* 3/1) from the solution at room temperature after two days.

### Refinement

All hydrogens were were calculated geometrically. The positions of the H atoms of the nitrogen atoms were refined using a riding model with N—H = 0.89 Å and *U*_iso_(H) = 1.5*U*_eq_(N). C—H groups were also refined using a riding model for hydrogen atoms with C—H distances ranging from 0.93 to 0.97 Å and *U*_iso_(H) = 1.2Ueq(C).

### Crystal data

**Table:**

(C_6_H_7_FN)[FeCl_4_]·C_12_H_24_O_6_	*F*(000) = 1188
*M**_r_* = 574.09	*D*_x_ = 1.430 Mg m^−^^3^
Monoclinic, *P*2_1_/*c*	Mo *K*α radiation, λ = 0.71073 Å
Hall symbol: -P 2ybc	Cell parameters from 5625 reflections
*a* = 11.45 (1) Å	θ = 2.3–27.5°
*b* = 24.14 (2) Å	µ = 1.00 mm^−^^1^
*c* = 9.719 (9) Å	*T* = 293 K
β = 96.82 (2)°	Block, pale yellow
*V* = 2667 (4) Å^3^	0.20 × 0.20 × 0.20 mm
*Z* = 4	

### Data collection

**Table:**

Rigaku SCXmini diffractometer	6039 independent reflections
Radiation source: fine-focus sealed tube	3173 reflections with *I* > 2σ(*I*)
graphite	*R*_int_ = 0.068
Detector resolution: 13.6612 pixels mm^-1^	θ_max_ = 27.5°, θ_min_ = 2.3°
ω scans	*h* = −14→14
Absorption correction: multi-scan (*CrystalClear*; Rigaku, 2005)	*k* = −31→31
*T*_min_ = 0.818, *T*_max_ = 0.818	*l* = −12→12
26978 measured reflections	

### Refinement

**Table:**

Refinement on *F*^2^	Primary atom site location: structure-invariant direct methods
Least-squares matrix: full	Secondary atom site location: difference Fourier map
*R*[*F*^2^ > 2σ(*F*^2^)] = 0.078	Hydrogen site location: inferred from neighbouring sites
*wR*(*F*^2^) = 0.271	H-atom parameters constrained
*S* = 1.07	*w* = 1/[σ^2^(*F*_o_^2^) + (0.1312*P*)^2^ + 0.8151*P*] where *P* = (*F*_o_^2^ + 2*F*_c_^2^)/3
6039 reflections	(Δ/σ)_max_ = 0.005
281 parameters	Δρ_max_ = 0.49 e Å^−^^3^
0 restraints	Δρ_min_ = −0.35 e Å^−^^3^

### Special details

**Table:**

Geometry. All esds (except the esd in the dihedral angle between two l.s. planes) are estimated using the full covariance matrix. The cell esds are taken into account individually in the estimation of esds in distances, angles and torsion angles; correlations between esds in cell parameters are only used when they are defined by crystal symmetry. An approximate (isotropic) treatment of cell esds is used for estimating esds involving l.s. planes.
Refinement. Refinement of *F*^2^ against ALL reflections. The weighted *R*-factor wR and goodness of fit *S* are based on *F*^2^, conventional *R*-factors *R* are based on *F*, with *F* set to zero for negative *F*^2^. The threshold expression of *F*^2^ > σ(*F*^2^) is used only for calculating *R*-factors(gt) etc. and is not relevant to the choice of reflections for refinement. *R*-factors based on *F*^2^ are statistically about twice as large as those based on *F*, and *R*- factors based on ALL data will be even larger.

### Fractional atomic coordinates and isotropic or equivalent isotropic displacement parameters (Å^2^)

**Table:**

	*x*	*y*	*z*	*U*_iso_*/*U*_eq_	
O1	0.5434 (4)	0.09467 (19)	0.7021 (5)	0.0842 (13)	
O2	0.7834 (4)	0.06611 (18)	0.6808 (4)	0.0816 (12)	
O3	0.9463 (3)	0.14900 (18)	0.7297 (5)	0.0741 (11)	
O4	0.8972 (4)	0.23864 (18)	0.8904 (5)	0.0849 (13)	
O5	0.6635 (4)	0.25943 (19)	0.9254 (5)	0.0903 (14)	
O6	0.4892 (4)	0.1787 (2)	0.8722 (5)	0.0895 (14)	
C1	0.5820 (8)	0.0397 (3)	0.6788 (9)	0.102 (2)	
H1A	0.5188	0.0190	0.6268	0.122*	
H1B	0.6025	0.0212	0.7670	0.122*	
C2	0.6847 (7)	0.0408 (3)	0.6013 (8)	0.093 (2)	
H2A	0.7049	0.0033	0.5776	0.112*	
H2B	0.6653	0.0613	0.5158	0.112*	
C3	0.8831 (6)	0.0647 (3)	0.6178 (7)	0.0800 (18)	
H3A	0.8688	0.0824	0.5278	0.096*	
H3B	0.9053	0.0265	0.6039	0.096*	
C4	0.9802 (6)	0.0937 (3)	0.7041 (7)	0.0798 (18)	
H4A	0.9987	0.0743	0.7913	0.096*	
H4B	1.0500	0.0939	0.6565	0.096*	
C5	1.0380 (5)	0.1806 (3)	0.7950 (8)	0.086 (2)	
H5A	1.1034	0.1806	0.7400	0.104*	
H5B	1.0650	0.1648	0.8850	0.104*	
C6	0.9964 (6)	0.2383 (3)	0.8120 (9)	0.091 (2)	
H6A	1.0596	0.2603	0.8595	0.109*	
H6B	0.9739	0.2546	0.7216	0.109*	
C7	0.8554 (8)	0.2921 (3)	0.9050 (10)	0.104 (3)	
H7A	0.8286	0.3074	0.8145	0.124*	
H7B	0.9180	0.3154	0.9488	0.124*	
C8	0.7560 (8)	0.2905 (3)	0.9917 (11)	0.113 (3)	
H8A	0.7825	0.2743	1.0812	0.136*	
H8B	0.7293	0.3280	1.0067	0.136*	
C9	0.5658 (7)	0.2584 (4)	0.9976 (9)	0.100 (2)	
H9A	0.5470	0.2955	1.0261	0.120*	
H9B	0.5813	0.2355	1.0798	0.120*	
C10	0.4667 (6)	0.2353 (3)	0.9035 (9)	0.091 (2)	
H10A	0.3948	0.2377	0.9469	0.109*	
H10B	0.4561	0.2567	0.8184	0.109*	
C11	0.4035 (6)	0.1551 (4)	0.7758 (10)	0.101 (2)	
H11A	0.3981	0.1759	0.6899	0.122*	
H11B	0.3276	0.1569	0.8108	0.122*	
C12	0.4324 (6)	0.0968 (3)	0.7491 (10)	0.100 (2)	
H12A	0.4329	0.0753	0.8334	0.119*	
H12B	0.3736	0.0813	0.6797	0.119*	
F1	0.8204 (4)	0.00256 (15)	0.3199 (4)	0.0906 (12)	
N1	0.7364 (3)	0.14716 (16)	−0.1213 (4)	0.0498 (9)	
H1C	0.7853	0.1760	−0.1140	0.075*	
H1D	0.6625	0.1592	−0.1276	0.075*	
H1E	0.7467	0.1277	−0.1967	0.075*	
C13	0.8711 (5)	0.0971 (3)	0.0489 (7)	0.0748 (17)	
H13A	0.9340	0.1117	0.0085	0.090*	
C14	0.7606 (4)	0.11179 (19)	0.0016 (5)	0.0493 (11)	
C15	0.6684 (5)	0.0924 (3)	0.0657 (7)	0.0699 (16)	
H15A	0.5920	0.1037	0.0355	0.084*	
C16	0.6894 (6)	0.0558 (3)	0.1752 (7)	0.0758 (17)	
H16A	0.6279	0.0427	0.2206	0.091*	
C17	0.8011 (6)	0.0396 (2)	0.2148 (6)	0.0658 (15)	
C18	0.8913 (6)	0.0598 (3)	0.1588 (7)	0.0804 (18)	
H18A	0.9676	0.0493	0.1922	0.096*	
Fe2	0.25883 (7)	0.12580 (3)	0.22601 (9)	0.0635 (3)	
Cl1	0.44359 (16)	0.11643 (9)	0.3030 (3)	0.1179 (8)	
Cl2	0.2109 (2)	0.06493 (9)	0.0642 (2)	0.1121 (7)	
Cl3	0.22522 (16)	0.20828 (7)	0.13775 (19)	0.0840 (5)	
Cl4	0.1561 (2)	0.11388 (10)	0.3981 (2)	0.1144 (8)	

### Atomic displacement parameters (Å^2^)

**Table:**

	*U*^11^	*U*^22^	*U*^33^	*U*^12^	*U*^13^	*U*^23^
O1	0.079 (3)	0.078 (3)	0.094 (3)	−0.018 (2)	0.006 (2)	−0.002 (2)
O2	0.104 (4)	0.073 (3)	0.070 (3)	0.002 (2)	0.015 (2)	−0.007 (2)
O3	0.058 (2)	0.079 (3)	0.086 (3)	0.010 (2)	0.012 (2)	0.006 (2)
O4	0.081 (3)	0.076 (3)	0.100 (3)	−0.021 (2)	0.021 (2)	−0.006 (2)
O5	0.086 (3)	0.078 (3)	0.109 (4)	0.010 (2)	0.022 (3)	−0.015 (3)
O6	0.062 (3)	0.098 (3)	0.109 (4)	0.013 (2)	0.013 (2)	0.017 (3)
C1	0.122 (7)	0.089 (5)	0.091 (5)	−0.017 (5)	0.003 (5)	−0.016 (4)
C2	0.115 (6)	0.074 (4)	0.090 (5)	−0.018 (4)	0.011 (4)	−0.024 (4)
C3	0.093 (5)	0.077 (4)	0.073 (4)	0.029 (4)	0.017 (3)	−0.005 (3)
C4	0.080 (4)	0.072 (4)	0.089 (5)	0.020 (3)	0.017 (4)	0.000 (3)
C5	0.045 (3)	0.101 (5)	0.114 (5)	−0.010 (3)	0.010 (3)	0.013 (4)
C6	0.068 (4)	0.097 (5)	0.110 (6)	−0.021 (4)	0.020 (4)	0.006 (4)
C7	0.127 (7)	0.066 (4)	0.120 (6)	−0.022 (4)	0.025 (5)	−0.029 (4)
C8	0.112 (6)	0.092 (5)	0.136 (7)	−0.022 (5)	0.017 (5)	−0.046 (5)
C9	0.091 (5)	0.109 (6)	0.108 (6)	0.024 (4)	0.044 (5)	0.002 (5)
C10	0.071 (4)	0.085 (5)	0.121 (6)	0.029 (4)	0.036 (4)	0.012 (4)
C11	0.057 (4)	0.117 (6)	0.129 (6)	−0.003 (4)	0.011 (4)	0.036 (5)
C12	0.059 (4)	0.102 (6)	0.135 (7)	−0.028 (4)	0.002 (4)	0.013 (5)
F1	0.122 (3)	0.087 (2)	0.065 (2)	0.032 (2)	0.019 (2)	0.0282 (19)
N1	0.048 (2)	0.049 (2)	0.053 (2)	0.0015 (17)	0.0086 (18)	0.0046 (18)
C13	0.053 (3)	0.096 (4)	0.075 (4)	−0.004 (3)	0.006 (3)	0.019 (3)
C14	0.054 (3)	0.048 (3)	0.047 (3)	0.002 (2)	0.011 (2)	−0.003 (2)
C15	0.053 (3)	0.078 (4)	0.081 (4)	0.012 (3)	0.018 (3)	0.022 (3)
C16	0.072 (4)	0.075 (4)	0.086 (4)	0.011 (3)	0.032 (3)	0.024 (3)
C17	0.090 (4)	0.055 (3)	0.053 (3)	0.007 (3)	0.010 (3)	0.005 (2)
C18	0.061 (4)	0.105 (5)	0.074 (4)	0.015 (3)	0.003 (3)	0.026 (4)
Fe2	0.0551 (5)	0.0653 (5)	0.0706 (5)	0.0049 (4)	0.0089 (4)	−0.0102 (4)
Cl1	0.0592 (10)	0.1006 (14)	0.187 (2)	0.0182 (9)	−0.0152 (12)	−0.0312 (14)
Cl2	0.1251 (17)	0.0938 (13)	0.1119 (15)	0.0013 (11)	−0.0090 (12)	−0.0418 (12)
Cl3	0.0859 (11)	0.0766 (10)	0.0914 (11)	0.0080 (8)	0.0180 (9)	0.0043 (9)
Cl4	0.1298 (18)	0.1178 (16)	0.1061 (15)	0.0209 (13)	0.0577 (13)	0.0211 (12)

### Geometric parameters (Å, °)

**Table:**

O1—C12	1.401 (8)	C8—H8B	0.9700
O1—C1	1.425 (9)	C9—C10	1.480 (12)
O2—C3	1.357 (8)	C9—H9A	0.9700
O2—C2	1.428 (8)	C9—H9B	0.9700
O3—C5	1.388 (7)	C10—H10A	0.9700
O3—C4	1.420 (7)	C10—H10B	0.9700
O4—C7	1.388 (8)	C11—C12	1.476 (11)
O4—C6	1.441 (8)	C11—H11A	0.9700
O5—C9	1.390 (8)	C11—H11B	0.9700
O5—C8	1.391 (9)	C12—H12A	0.9700
O6—C11	1.396 (9)	C12—H12B	0.9700
O6—C10	1.428 (8)	F1—C17	1.356 (6)
C1—C2	1.471 (11)	N1—C14	1.467 (6)
C1—H1A	0.9700	N1—H1C	0.8900
C1—H1B	0.9700	N1—H1D	0.8900
C2—H2A	0.9700	N1—H1E	0.8900
C2—H2B	0.9700	C13—C14	1.341 (8)
C3—C4	1.486 (10)	C13—C18	1.396 (8)
C3—H3A	0.9700	C13—H13A	0.9300
C3—H3B	0.9700	C14—C15	1.371 (7)
C4—H4A	0.9700	C15—C16	1.381 (8)
C4—H4B	0.9700	C15—H15A	0.9300
C5—C6	1.487 (10)	C16—C17	1.349 (9)
C5—H5A	0.9700	C16—H16A	0.9300
C5—H5B	0.9700	C17—C18	1.317 (8)
C6—H6A	0.9700	C18—H18A	0.9300
C6—H6B	0.9700	Fe2—Cl1	2.170 (3)
C7—C8	1.495 (12)	Fe2—Cl2	2.175 (2)
C7—H7A	0.9700	Fe2—Cl4	2.175 (3)
C7—H7B	0.9700	Fe2—Cl3	2.184 (2)
C8—H8A	0.9700		
			
C12—O1—C1	113.4 (6)	O5—C9—C10	107.4 (7)
C3—O2—C2	113.5 (5)	O5—C9—H9A	110.2
C5—O3—C4	112.9 (5)	C10—C9—H9A	110.2
C7—O4—C6	111.2 (5)	O5—C9—H9B	110.2
C9—O5—C8	113.0 (7)	C10—C9—H9B	110.2
C11—O6—C10	113.7 (6)	H9A—C9—H9B	108.5
O1—C1—C2	110.2 (6)	O6—C10—C9	110.3 (6)
O1—C1—H1A	109.6	O6—C10—H10A	109.6
C2—C1—H1A	109.6	C9—C10—H10A	109.6
O1—C1—H1B	109.6	O6—C10—H10B	109.6
C2—C1—H1B	109.6	C9—C10—H10B	109.6
H1A—C1—H1B	108.1	H10A—C10—H10B	108.1
O2—C2—C1	111.2 (6)	O6—C11—C12	110.7 (6)
O2—C2—H2A	109.4	O6—C11—H11A	109.5
C1—C2—H2A	109.4	C12—C11—H11A	109.5
O2—C2—H2B	109.4	O6—C11—H11B	109.5
C1—C2—H2B	109.4	C12—C11—H11B	109.5
H2A—C2—H2B	108.0	H11A—C11—H11B	108.1
O2—C3—C4	110.3 (6)	O1—C12—C11	108.9 (6)
O2—C3—H3A	109.6	O1—C12—H12A	109.9
C4—C3—H3A	109.6	C11—C12—H12A	109.9
O2—C3—H3B	109.6	O1—C12—H12B	109.9
C4—C3—H3B	109.6	C11—C12—H12B	109.9
H3A—C3—H3B	108.1	H12A—C12—H12B	108.3
O3—C4—C3	109.9 (5)	C14—N1—H1C	109.5
O3—C4—H4A	109.7	C14—N1—H1D	109.5
C3—C4—H4A	109.7	H1C—N1—H1D	109.5
O3—C4—H4B	109.7	C14—N1—H1E	109.5
C3—C4—H4B	109.7	H1C—N1—H1E	109.5
H4A—C4—H4B	108.2	H1D—N1—H1E	109.5
O3—C5—C6	109.3 (5)	C14—C13—C18	119.8 (5)
O3—C5—H5A	109.8	C14—C13—H13A	120.1
C6—C5—H5A	109.8	C18—C13—H13A	120.1
O3—C5—H5B	109.8	C13—C14—C15	120.0 (5)
C6—C5—H5B	109.8	C13—C14—N1	120.8 (5)
H5A—C5—H5B	108.3	C15—C14—N1	119.2 (5)
O4—C6—C5	110.3 (5)	C14—C15—C16	119.7 (5)
O4—C6—H6A	109.6	C14—C15—H15A	120.1
C5—C6—H6A	109.6	C16—C15—H15A	120.1
O4—C6—H6B	109.6	C17—C16—C15	118.5 (5)
C5—C6—H6B	109.6	C17—C16—H16A	120.7
H6A—C6—H6B	108.1	C15—C16—H16A	120.7
O4—C7—C8	109.2 (7)	C18—C17—C16	122.6 (6)
O4—C7—H7A	109.8	C18—C17—F1	119.3 (6)
C8—C7—H7A	109.8	C16—C17—F1	118.1 (6)
O4—C7—H7B	109.8	C17—C18—C13	119.2 (6)
C8—C7—H7B	109.8	C17—C18—H18A	120.4
H7A—C7—H7B	108.3	C13—C18—H18A	120.4
O5—C8—C7	109.9 (7)	Cl1—Fe2—Cl2	109.35 (9)
O5—C8—H8A	109.7	Cl1—Fe2—Cl4	108.36 (13)
C7—C8—H8A	109.7	Cl2—Fe2—Cl4	110.70 (12)
O5—C8—H8B	109.7	Cl1—Fe2—Cl3	110.46 (9)
C7—C8—H8B	109.7	Cl2—Fe2—Cl3	108.32 (11)
H8A—C8—H8B	108.2	Cl4—Fe2—Cl3	109.66 (8)

### Hydrogen-bond geometry (Å, °)

**Table:**

*D*—H···*A*	*D*—H	H···*A*	*D*···*A*	*D*—H···*A*
N1—H1C···O4^i^	0.89	1.98	2.868 (6)	176
N1—H1D···O6^i^	0.89	2.04	2.924 (6)	173
N1—H1E···O2^i^	0.89	1.98	2.840 (6)	162

Symmetry codes: (i) *x*, *y*, *z*−1.
